# Imaging Strategies in Proton Therapy for Thoracic Tumors: A Mini Review

**DOI:** 10.3389/fonc.2022.833364

**Published:** 2022-04-14

**Authors:** Carlo Algranati, Lidia Strigari

**Affiliations:** ^1^ Proton Therapy Department, Azienda Provinciale per i Servizi Sanitari (APSS), Trento, Italy; ^2^ Dipartimento di Medicina Specialistica, Diagnostica e Sperimentale (DIMES), University of Bologna, Bologna, Italy; ^3^ Department of Medical Physics, Istituto di Ricovero e Cura a Carattere Scientifico (IRCCS) Azienda Ospedaliero-Universitaria di Bologna, Bologna, Italy

**Keywords:** proton therapy, imaging, thoracic tumors, motion management in radiotherapy, adaptive, radiotherapy

## Abstract

Proton beam therapy (PBT) is often more attractive for its high gradient dose distributions than other treatment modalities with external photon beams. However, in thoracic lesions treated particularly with pencil beam scanning (PBS) proton beams, several dosimetric issues are addressed. The PBS approach may lead to large hot or cold spots in dose distributions delivered to the patients, potentially affecting the tumor control and/or increasing normal tissue side effects. This delivery method particularly benefits image-guided approaches. Our paper aims at reviewing imaging strategies and their technological trends for PBT in thoracic lesions. The focus is on the use of imaging strategies in simulation, planning, positioning, adaptation, monitoring, and delivery of treatment and how changes in the anatomy of thoracic tumors are handled with the available tools and devices in PBT. Starting from bibliographic research over the past 5 years, retrieving 174 papers, major key questions, and implemented solutions were identified and discussed; the results aggregated and presented following the methodology of analysis of expert interviews.

## 1 Introduction

Radiation therapy (RT) is an essential and effective tool in the curative treatment of different anatomical sites. Unfortunately, with photon RT, safe dose escalation, delivery of concomitant systemic therapy, or re-irradiation of the recurrent disease may not be feasible due to radiation-induced toxicities. In contrast, the finite range of proton beams in tissues offers unique dosimetric advantages that theoretically allow escalating the target dose, potentially prolonging survival while minimizing exposure of surrounding tissues and consequently radiation-induced toxicity rate.

This theoretical advantage has led to the widespread adoption of proton beam therapy (PBT) worldwide for a wide variety of thoracic malignancies, including lung cancer, esophageal cancer, mesothelioma, and thymic cancer. At the state of the art, the tremendous potential of PBT for treating thoracic cancers is only beginning to be appreciated.

PBT provided a lower total toxicity burden, particularly pulmonary, cardiac, and hematologic toxicity, within the context of previous attempts at dose escalation for lung and esophageal cancer (1). Similarly, for mesothelioma patients, the physical properties of proton therapy result in better sparing of normal tissues, particularly in treating the pleura, in both post-pneumonectomy and lung-intact settings. There are drastic dose reductions to the contralateral lung, heart, liver, kidneys, and stomach (2). Re-irradiation, advanced disease requiring extensive cardio-pulmonary irradiated volumes, and younger patients may likely benefit from modern PBT (3). New techniques like stereotactic body radiation therapy (SBRT) and PBT are now increasingly adopted as the only radical treatment for small solitary lung tumors (4) and represent the most used non-surgical modality in treating lung cancers, permitting the improvement of treatment outcomes and favorable toxicities.

Moreover, treating thoracic cancers involves solving most technical and technological imaging, treatment plan, delivery, and adaptive problems. Several issues are relevant for improving the efficacy and safety of thoracic moving tumors or tumor shrinkage/anatomical changes during the treatment, such as the type of online imaging and the vulnerability of protons to inherent heterogeneities in the beam path. Therefore, there is an enhancing need to perform adaptive planning, representing the key to more comprehensive PBT application (1).

New approaches to combining PBT and immunotherapy (5) demand creative investigation for introducing ultrahigh dose-rate Flash, GRID/lattice, and microbeam delivery approaches in PBT (6–8). The maturity of technologies, including treatment planning and image-guided technology, is the critical issue for realizing new PBT treatment strategies.

This work focuses on imaging and motion-related devices used for PBT treatment simulation, planning, positioning, adaptation, monitoring, and delivery in thoracic tumors.

## 2 Materials and Methods

### 2.1 Literature Search Strategy

A PubMed search was performed using the query string to identify the publication related to proton therapy in thoracic tumors, mainly represented by small-cell lung cancer (SCLC) and non-small cell lung cancer (NSCLC), mesothelioma, thymoma, and esophageal cancer. These thoracic malignancies are challenging from the treatment point of view because of relevant tissue heterogeneities, the presence of moving organs and targets, and the limited availability of onboard soft tissue imaging devices.

We included the following keywords/strings in the PubMed query search:

•“proton therapy” AND “thoracic”;
•“proton therapy” AND “non-small-cell-lung-cancers”;
•“proton therapy” AND “small-cell-lung-cancer”;
•“proton therapy” AND “mesothelioma”;
•“proton therapy” AND (“thymoma” OR “thymic malignancy”);
•“proton therapy” AND “esophageal cancer”.

Filters are from June 3, 2016 to June 3, 2021. The research was restricted to the last 5 years to include only the keywords in the title and/or abstract. The search was done on the June 3, 2021.

### 2.2 Study Selection

Two authors independently reviewed titles and abstracts to decide the study inclusion. Full articles were retrieved when the abstract was considered relevant. Only papers or abstracts published in English were considered.

Papers were selected if they contained information about the treatment of thoracic tumors with PBT and gave answers or inside view on the following medical physics questions:

1. Which is the imaging approach for simulation, planning, positioning, adaptation, monitoring, and delivery in PBT for thoracic tumors?
2. How are handled changes in the anatomy of thoracic tumors with the available tools and devices in PBT?

The data were summarized in a database with the following issues: first author, journal, year, title.

Data analysis and interpretation rely on Bogner and Menz’s (9) related to Expert interviews.

## 3 Results

### 3.1 Description of Included Studies and Inclusion Criteria

**Figure 1:**
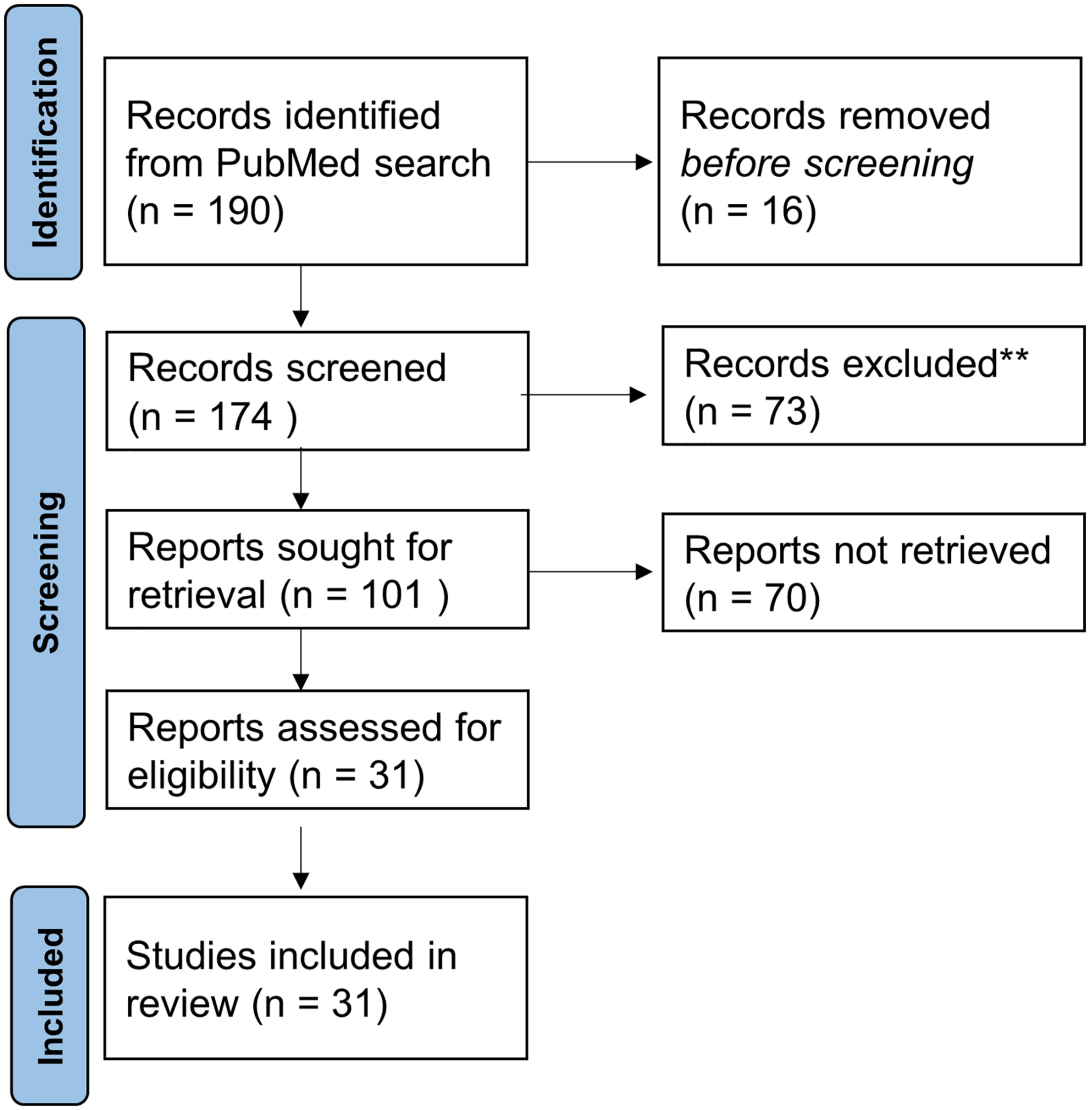
Numbers of included and excluded papers derived from the PubMed search related to PBT in thoracic tumors.

Based on the reported PubMed/Medline search, 190 papers and abstracts were identified. The results are represented in **Figure 1**. Substantial growth was observed looking at the included papers through the years. The number of papers related to 2021 was not complete because the inclusion criteria were limited to June 3, 2021.

Out of the 190 records, 16 were excluded as duplicates. 73 papers of 174 records screened were excluded for the following reasons: not inherent (#73) to the addressed questions. Out of the 102 full-text articles assessed for eligibility, 31 full-text articles were selected according to medical physics-related questions 1 and 2 described in paragraph study selection in *Material and Methods*.

**Figure 2:**
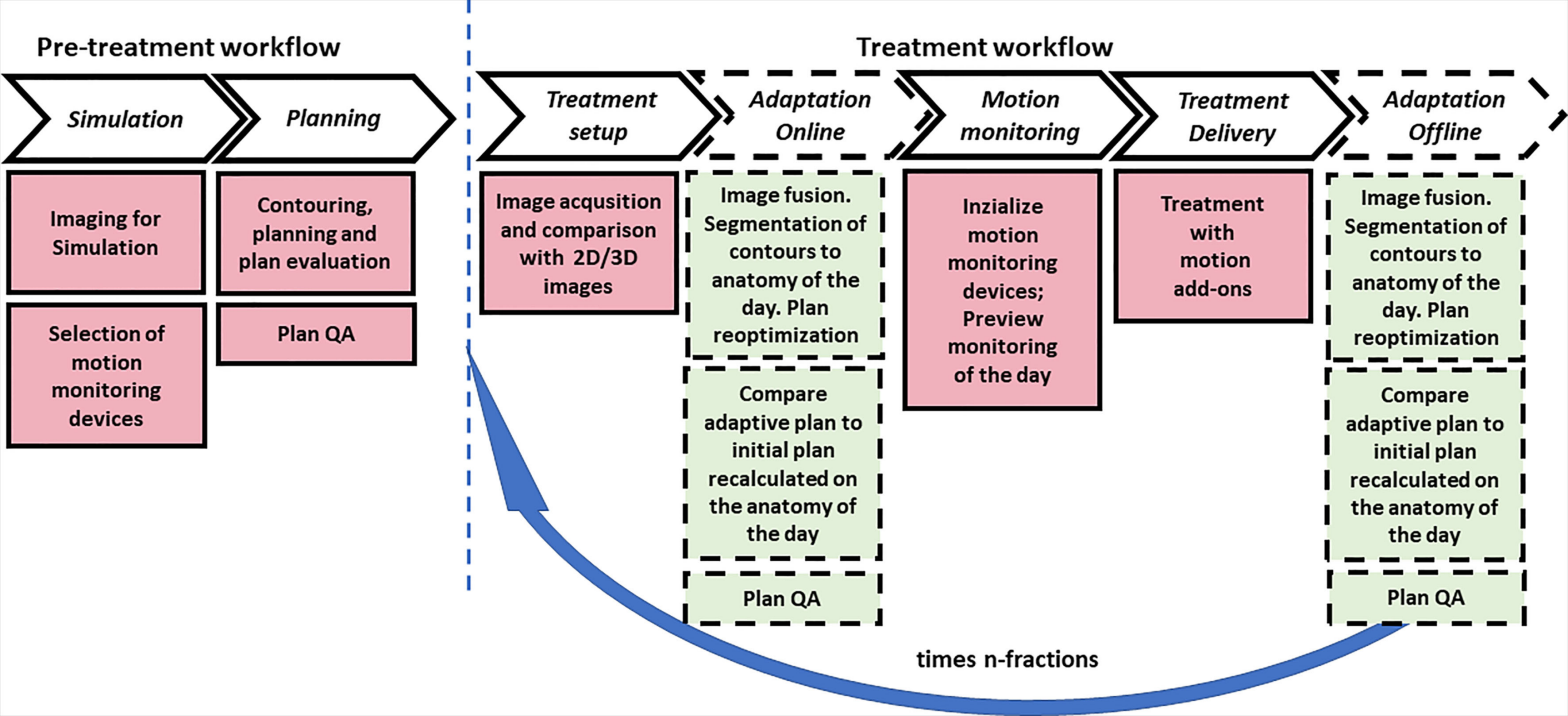
Typical pretreatment and treatment PBT workflow with main subitems/tasks for motion management and anatomical change of organs and tumors. The dashed blocks indicate the online and off-line adaptation tasks that may be optionally applied one or more times during treatment. The blue arrow indicates the number of fractions in which the inter- and intra-fraction evaluation for motion management is applied.

The following groups were obtained according to the typical PBT workflow phases in a clinic (see **Figure 2**): simulation (# 24), planning (#9), treatment setup (#13), adaptation (#8), motion monitoring (#6), and treatment delivery (#6). Papers that give information about two or more groups were counted once for each group.

Additional subgroups related to subphases are identified in **Figure 2** as reported in *Results*.

### 3.2 Simulation

In thoracic tumors, if necessary, respiratory-gated or breath-hold radiation therapy techniques are used to accommodate tumor motion; however, most patients are treated with free-breathing, which is the more efficient technique (10). According to this, we have reported simulation imaging approaches and motion handling methods in agreement with the international guidelines in particle therapy thoracic malignancies (11).

#### 3.2.1 Setup

Patients are mostly immobilized supine in an upper-body cradle with arms overhead (12). A small Vac-Lok cushion is used on a so-called wing board to stabilize the arms and the head. The pelvis and legs are stabilized with larger vacuum bags or directly positioned on the couch. Patients lay on the table with a gap between the head and pelvic vacuum bags to minimize material in the beam path (13, 14). The type of cushions is critical because they could introduce uncertainties in the alignment of different tissues along the beam path (15).

Significant internal motion is accounted for by utilizing various measures, including 4-dimensional (4D) CT imaging and abdominal compression, and/or through the placement of internal fiducials at the discretion of the treating radiation oncologist. Sometimes abdominal compression devices are reported (16).

#### 3.2.2 Imaging for Simulation

The free-breathing patients underwent CT simulation with 4D-CT to account for respiratory motion with deformation (12, 15, 17–24). Regarding the motion assessment, a tumor motion range less than 5 mm is considered acceptable for free-breathing delivery (15). Patient respiratory waveforms were monitored throughout the procedures and recorded with a respiratory gating system (14, 17, 19, 20, 25–27). Alternatively, to mitigate intra-fractional motion in NSCLC patients, visually guided voluntary DIBH CT images are acquired (26, 28–32).

### 3.3 Planning

#### 3.3.1 Contouring and Plan Evaluation

The use of ^18^F-fluorodeoxyglucose-PET/computed tomography (CT) is well established in lung cancer and several other thoracic malignancies. Simone et al. (33) describes the expected future roles of PET/CT for thoracic tumors. In the free-breathing cases, the gross tumor volume (GTV) is often defined based on all available clinical information in the average intensity projection reconstruction images derived from all breathing phases of the 4D-CT (19–21, 25, 34). The plan is then generated on an averaged 4D-CTs with possible density overwrites. The method reported by Fracchiolla et al. (23) is to create internal target volume (ITV) on the free-breathing CT as the union of all CTVs contoured on each phase of the 4D-CT. The plan is calculated on the free-breathing CT.

#### 3.3.2 Range Uncertainty

In selecting imaging CT for planning, the uncertainty related to the proton-stopping-power conversion of the Hounsfield units plays an essential role. Currently, algorithms using single energy CT photon to proton stopping power calculations implement a 3%–3.5% uncertainty for each centimeter (cm) of beam path length (15). However, for planning purposes, one technical advance that undoubtedly may improve proton treatment for NSCLC is the employment of dual-energy CT (DECT) or other techniques that reduce range uncertainty for treatment delivery (35, 36).

#### 3.3.3 Special Approaches

Incorporating 4D-CT ventilation imaging into functional proton therapy is feasible (37). In intensity-modulated proton delivery, the functional proton plans are adequate to further preserve high-functioning lung regions without degrading the PTV coverage. This approach is feasible in a subset of patients with breathing motion limited to 5–7 mm from CT0 (inhalation) to CT50 (max exhalation) (25, 37).

Sala et al. (32) propose high-frequency percussive ventilation (HFPV) to reduce motion impact drastically. This approach employs high-frequency low tidal volumes (100–400 bursts/min) to provide respiration in awake patients.

#### 3.3.4 Motion Monitor Devices

Different motion monitoring devices are reported used in combination with X-ray imaging devices adopted for planning, setup, and delivery in PBT. They are classifiable according to the type of implemented technology in surface-guided radiotherapy (SGRT) using optical systems, spirometry, and markers.

The SGRT systems include commercial ones such as the Varian Real-time Position Management system (Varian Medical Systems, Palo Alto, CA) (19, 37), Vision RT (23), or in-house solutions (10). The spirometric systems included DIBH using the SDX system (Dyn’R-SDX, version 2.06) (30) and the Active Breathing Coordinator (ABC, Elekta Oncology Systems Ltd., Crawley, West Sussex, UK) (23). Several authors report using gold fiducial markers implanted by bronchoscopy (27, 28) or endobronchial ultrasound guidance (13). The patient’s respiratory stability was evaluated by studying the marker motion as a surrogate of tumor displacement using X-ray imaging devices. Elhammali et al. (12) reported that a minimum of 3 days for a trans-thoracic approach or 2 days for a bronchoscopy approach were required to allow fiducials to stabilize before simulation. Another reported system is the Z-733 V respiratory gating system (Anzai Medical, Tokyo, Japan) (17, 20, 22, 36, 38, 39).

### 3.4 Treatment Setup

#### 3.4.1 Image Acquisition and Comparison With 2D/3D Images

For standard treatments in patients with thoracic tumors, daily patient alignments are achieved by matching fiducial markers or vertebral bones with 2D/2D matching methods (10, 12, 13, 16, 17, 21, 27, 28, 34, 40). The setup is continued until the eventual fiducial markers on the digitally reconstructed radiographs are agreed within 2 mm (13, 28).

The 2D/2D fusion approach limits the visibility of soft tissues that is crucial for PBT beams and ensures adequate treatment plan delivery (21). The number of pencil beam scanned proton therapy (PBSPT) facilities equipped with cone-beam computed tomography (CBCT) imaging treating thoracic indications is constantly rising (34, 39, 40), thus allowing the implementation of dose summation and adaptive treatments. To overcome the absence of onboard 3D images, weekly CT images are acquired to generate verification and adaptive plans (18, 23).

### 3.5 Treatment Adaptation

Forsthoefel et al. (16) reported that treatment setup and delivery are verified with regularly scheduled quality assurance CT scans during treatment. Kharod et al. (13) reported that patients underwent verification CT scans on days 1, 2, 4, and 6 of treatment to confirm appropriate alignment. Chen et al. (41) did analyze the correlation between anatomic change and the need to adopt adaptive radiotherapy (ART).

#### 3.5.1 Off-Line Adaptive

Iwata et al. (28) reported that CT permits evaluating tumor shrinkage at the end of PBT. Replanning was conducted if beam leakage to the distal side due to tumor volume change (shrinkage) or body mass reduction was significant. The adaptive replanning was performed when the esophagus and spinal cord dose increased and exceeded the limit dose and/or when the dose to the lung adjacent to the tumor increased by about 10%.

#### 3.5.2 Online Adaptive

The online adaptive protocol was reported by (29) based on plan re-optimization using a fast but limited accuracy analytical algorithm that can still improve the overall treatment dose for patients with cancer in the lung and HN regions. Nevertheless, no online adaptive protocol was reported in the literature in the papers selected for this study.

### 3.6 Motion Monitoring and Treatment Delivery

In thoracic tumors, respiratory-gated PBT combined with image-guided techniques enables adaptive plan implementation (28). Abdominal surface motion is used as a surrogate for tumor motion, and the beam is turned on only when the monitored respiratory phase falls within the predefined gating window (17).

## 4 Discussion and Conclusions

To fully realize the potential of PBT for thoracic cancer, extensive improvements are needed in all the image-related aspects of the treatment process, from simulation, planning algorithms, and volumetric image guidance to real-time tracking and treatment adaptation.

For complicated anatomy, intensity-modulated PBT should be considered with appropriate motion management (27). For centrally located lesions and re-irradiation, volumetric imaging is crucial for accurate delivery and reducing the PTV margins.

Our review of selected literature highlighted some barriers for treating moving targets with significant tissue heterogeneity and the technologic efforts underway to overcome these challenges for thoracic malignancies. One of the most important of these was the lack of 3D volumetric imaging in the PBT facilities for treatment setup and adaptation. Because visualizing tumors with non-volumetric 2-dimensional images is challenging for PBT, fiducial markers are frequently adopted, although they represent an invasive procedure and are not always feasible. When tumors are close to bony structures, these could be used as a landmark (11).

In PBT, 4D-CT, ITV generation, and free-breathing are frequently reported approaches preferred to DIBH. The free-breathing approach applies mainly when the tumor displacements are limited up to 5 mm.

SGRT PBT is a reported option based on repeated CBCT or CT analysis. In photon beam radiotherapy, the availability of 4D-CBCT and 4D-CT allows assessing the correlation between the tumor/hepatic dome and skin displacements, enabling an appropriate intra- and inter-fraction motion management (42). The spirometry represents the most extensively adopted solution in PBT.

DECT is considered of interest for a more precise and accurate estimation of range uncertainties but is not currently clinically applied.

Adopting an online adaptive strategy demands onboard setup imaging (CBCT for generating synthetic CT) or CT on-rail and dedicated software, which permits the adaptation and an *in silico* plan quality assurance.

## Author Contributions

LS and CA designed the study. CA performed the PubMed search. LS and CA independently reviewed the titles and abstracts to decide the study inclusion, discussed the results, and contributed to the final manuscript. All authors contributed to the article and approved the submitted version.

## Conflict of Interest

The authors declare that the research was conducted in the absence of any commercial or financial relationships that could be construed as a potential conflict of interest.

## Publisher’s Note

All claims expressed in this article are solely those of the authors and do not necessarily represent those of their affiliated organizations, or those of the publisher, the editors and the reviewers. Any product that may be evaluated in this article, or claim that may be made by its manufacturer, is not guaranteed or endorsed by the publisher.
